# Explicit and Implicit Basic Human Motives, and Public Service Motivation

**DOI:** 10.3389/fpsyg.2020.01542

**Published:** 2020-06-30

**Authors:** Hendrik Slabbinck, Arjen Van Witteloostuijn

**Affiliations:** ^1^Department of Marketing, Innovation and Organisation, Ghent University, Ghent, Belgium; ^2^School of Business and Economics, Vrije Universiteit Amsterdam, Amsterdam, Netherlands; ^3^Antwerp Management School, University of Antwerp, Antwerp, Belgium; ^4^Department of Management, University of Antwerp, Antwerp, Belgium

**Keywords:** Brief Implicit Association Test, public service motivation, implicit motives, personality, implicit measures

## Abstract

This article contributes to the literature on the roots of Public Service Motivation (PSM) by turning to the psychological theory of basic human motives. The study explores the differential associations of explicit and implicit basic human motives with PSM, Attraction to Policy-Making (APM), Commitment to the Public Interest (CPI), Compassion (COM), and Self-Sacrifice (SS). Methodologically, the research contributes to the literature by introducing a measurement instrument new to Public Administration: the Brief Implicit Association Test (BIAT). The BIAT is an easy-to-use and flexible tool to probe into the human unconsciousness, offering ample opportunities for further research in Public Administration and Management.

## Introduction

Research on Public Service Motivation (PSM) has grown exponentially in recent decades (Ritz et al., 2016; Christensen et al., 2017). PSM relates to the beliefs, values and attitudes that go beyond self-interest and organizational interest, reflecting someone’s willingness to help society and its citizens (Perry and Wise, 1990; Kim and Vandenabeele, 2010). Not surprisingly, given this definition, PSM is argued to be a driver to become a civil servant, and to subsequently flourish as a public sector employee (Vandenabeele, 2008; Christensen et al., 2017), but the concept certainly extends beyond public sector employees. Also students, private sector employees, entrepreneurs and citizens may feel themselves motivated to serve the public interest (Christensen et al., 2017).

High PSM can be beneficial for both individuals and their societies (Harari et al., 2017). For example, higher PSM is associated with higher job satisfaction (Vandenabeele, 2007; Giauque et al., 2012; Kim, 2012), increased organizational commitment (Levitats and Vigoda-Gadot, 2017), prosocial behavior (Esteve et al., 2016), organizational citizenship behavior (Bottomley et al., 2016; Esteve et al., 2016), and innovative behavior (Miao et al., 2018). Hence, gaining better insights into the formation of PSM is important. Prior work has identified many antecedents of PSM (for an overview, see Perry et al., 2008), most studies focusing on socio-demographic or vocational variables (Perry et al., 2008; Ritz et al., 2016). Public Administration literature certainly acknowledges the importance of deeper, more fundamental individual differences, but research on the underlying psychological antecedents of PSM is very scarce (Charbonneau and Van Ryzin, 2017; van Witteloostuijn et al., 2017). Recently, van Witteloostuijn et al. (2017) explored the connection between personality traits and PSM, reporting that a person’s personality profile is strongly connected to PSM. This article takes a next step by connecting human motives theory to PSM.

Human personality consists of many more constructs than just personality traits (Buss and Cantor, 1989). Next to traits, personality research devoted much attention to human motives (Buss and Cantor, 1989; Emmons, 1989). Psychology’s human motives theory distinguishes three fundamental human motives that are assumed to energize and drive behavior: the affiliation, power, and achievement need (McClelland et al., 1989; Schultheiss and Brunstein, 2010). Human motives theory is promising in an PSM context for two important reasons. Firstly, PSM is defined as a context-specific expression of prosocial behavior, grounded in the desire or the basic need to help others through public institutions (Perry and Wise, 1990; Kim and Vandenabeele, 2010). Interestingly, extant Psychology work reports strong connections between basic human motives and prosocial behavior (Hofer and Chasiotis, 2003; Hofer et al., 2007; Aydinli et al., 2014, 2016; Hermans et al., 2017).

Secondly, a long history of basic human motives research shows that people have conscious or explicit motives, as well as unconscious or implicit ones. Explicit motives involve controlled or conscious information processing and propositional reasoning, while implicit motives concern automatic or non-conscious information processing (McClelland et al., 1989). Current research relating psychological constructs to PSM has relied on explicit personality only (van Witteloostuijn et al., 2017), despite the fact that both explicit and implicit personality aspects operate independently from each other, each influencing different types of behavior (Perugini et al., 2010). Hence, the independent role of implicit motives is also considered here.

Thus, relying on McClelland’s human motives theory, our study aims to assess if basic human explicit and implicit motives are related to PSM, and how explicit and implicit motives differ in their relation to PSM. As PSM is often considered as a multi-facetted construct (Perry, 1996; Kim, 2011), with compassion, self-sacrifice, attraction to policy-making, and commitment to the public interest as its subdimensions, we also investigate how explicit and implicit motives are connected to each of these facets of PSM.

Accordingly, our research contributes to the PSM literature in at least three ways. First, by introducing basic human motives as another part of a human’s personality, the theoretical foundation of the PSM construct is strengthened, answering to the call to employ psychological theories to advance our understanding of important societal phenomena (van Witteloostuijn et al., 2017). Second, the study presents a completely new class of antecedents: implicit human motives. Indeed, the results show that PSM is influenced by both the explicit and implicit motives of an individual, but differently so. Third, as implicit concepts require different measurement techniques, implying that we introduce a new class of assessment techniques to the PSM community and PA at large.

## Theory

### Explicit and Implicit Motives

The individual’s socio-historical background (such as education and income) and motivational context (e.g., institutional characteristics and cultural differences) are the two sources traditionally assumed to affect PSM (Perry, 2000). van Witteloostuijn et al. (2017) were the first to evaluate the psychological antecedents of PSM. Relying on the HEXACO personality model (Ashton and Lee, 2001), they showed that PSM is strongly influenced by core personality traits. On the one hand, the more affective facets of PSM, Compassion and Self-Sacrifice, are positively associated with the personality traits of Honesty-Humility, Emotionality, and Agreeableness, and negatively with Conscientiousness. On the other hand, the non-affective facets of PSM, Attraction to Policy-Making and Commitment to the Public Interest, are positively correlated with Openness to Experience. Their models explains 5–15% of variance in affective, non-affective, and overall PSM. Although these percentages are perfectly in line with other studies linking personality and motivation (Furnham et al., 2009), this implies that most of the variance in PSM across individuals still remains to be explained.

To this end, we will explore McClelland’s basic human motives (McClelland et al., 1989). Indeed, human personality consists of many more constructs than just personality traits. Basic human motives, traits, values, wishes, attitudes, goals and beliefs represent a mere sampling of the conceptual units that have been employed in the pursuit of understanding the human personality (Buss and Cantor, 1989). Of these, basic human motives and traits have clearly received the greatest share of attention (Buss and Cantor, 1989; Emmons, 1989). Compared to personality traits, the human motives perspective represents a distinct framework to describe individual differences in personality (Winter et al., 1998; McAdams and Olson, 2010). Traits primarily refer to habitual patterns of *how* people think, feel, and act, whereas the human motives perspective focuses on *why* people do so. Human motives are defined as someone’s capacity to experience a specific type of stimulus, incentive or activity as pleasurable (Schultheiss and Brunstein, 2010), and refer to stable differences in classes of goals and desires from which people derive pleasure and satisfaction (McClelland et al., 1989). Human motives rather refer to individual goals, being distinct from traits that instead involve habitual patterns of behavior, ignoring why individuals show this behavior (Winter et al., 1998; Lang et al., 2012).

For example, Winter et al. (1998) suggest that extraverts might attend parties (i.e., personality-behavior link) to fulfill affiliative motives (i.e., human motive-goal link), whereas introverts may do so (personality-behavior link) to pursue other goals, such as networking for a job (i.e., human motive-goal link). This and other research (e.g., Chen et al., 2015; Hofer et al., 2015) indicates that human motives and personality traits connect to different levels or aspects of a person that may conjointly shape that person’s thoughts and feelings, interactively predicting the person’s behavior over the course of many years. Hence, next to personality traits, human motives may add to the understanding of the individual roots of PSM.

McClelland’s human motives model distinguishes three major motives: the need for achievement, affiliation, and power. The power motive stems from a person’s desire to influence, teach or encourage others. Individuals with a high need for power obtain satisfaction from exerting social, physical or emotional impact on others or on the world at large, but they experience social defeats and impacts from others as aversive (Winter, 1973). People with a high need for achievement typically get satisfaction from mastering challenging tasks on their own, but experience the failure to master such tasks individually as dissatisfying (McClelland et al., 1976). Finally, people who are motivated by affiliation prefer to spend time with others they like. They love to create, maintain, and restore social relationships. They enjoy being a part of a group, and have a desire to feel loved and accepted. Signals of rejection or hostility are experienced as unpleasant (Heyns et al., 1958).

Human motives theory distinguishes between two types of motivational systems–an implicit system that operates outside of conscious awareness and control, and an explicit system that functions at a conscious level (McClelland et al., 1989). Implicit motives differ fundamentally from explicit motives in that the former are acquired during early childhood on the basis of non-verbal, affective experiences, while the latter are cognitively more elaborated constructs that are acquired after the development of language, being influenced by explicit instructions originating in the social and cultural environment (McClelland and Pilon, 1983; Kasser et al., 2002). Using Berlew’s push–pull metaphor, Kehr (2004) and Hermans et al. (2017) suggest that implicit motives “push” individuals toward actions that they enjoy (“want-to” behavior), while explicit motives “pull” them toward actions that they feel obliged to do (“have-to” behaviors). Explicit and implicit motives are also triggered by different types of incentives, and they only affect behavior in the presence of the “right” type of incentive (McClelland et al., 1989; Stanton et al., 2010). In essence, implicit motives are triggered by task-intrinsic incentives, whereas explicit motives are aroused by social-extrinsic incentives. A task-intrinsic incentive relates to all aspects of a task or behavior that are inherently rewarding for an individual, whilst social-extrinsic incentives refer to social rewards and explicit instructions (Stanton et al., 2010). McClelland et al. (1989) also argued that social-extrinsic incentives do not trigger implicit motives, and that task-intrinsic incentives do not trigger explicit motives.

Implicit and explicit human motives also differ in their behavioral impact. Implicit motives associate with spontaneous, uncontrolled behavior, and effort-related task performance, whereas explicit human motives predict behavior that is subject to conscious thought and deliberation, such as self-reflective appraisals, judgments, and deliberate choices (Perugini et al., 2010; Schultheiss and Brunstein, 2010). Given the conscious representation of the explicit system, explicit human motives can be easily assessed via self-reports through questionnaires. In contrast, because implicit human motives operate outside of a person’s awareness, and since people often lack direct introspective insight into their implicit system, sensitive implicit measurement instruments are needed to measure these hidden individual differences (Schultheiss and Pang, 2007; Pang, 2010; Slabbinck et al., 2011).

Research on dispositional antecedents of PSM is so far exclusively based on explicit traits and motives, and neglects the influence of unconscious, implicit variables. Hence, research on the origin of PSM can expand toward the examination of implicit antecedents. In this study, we will assess both implicit and explicit human motives, using implicit tests and explicit self-reports, respectively. The exclusive reliance on self-reports restricts our understanding of the source of PSM to information that an individual can consciously access and is willing to report. Implicit measures can go beyond these restrictions (Greenwald et al., 1998; De Houwer et al., 2009; Gawronski and De Houwer, 2014).

### Public Service Motivation

Public Service Motivation (PSM) refers to the motives that individuals have to display behavior that they believe will have a positive influence on the public interest (Perry and Wise, 1990; Kim and Vandenabeele, 2010). PSM is often associated with four dimensions (Perry, 1996; Kim, 2011): Attraction to Policy-Making (APM), Commitment to the Public Interest (CPI), Compassion (COM), and Self-Sacrifice (SS). APM describes the degree to which people are dedicated to public service, community building, and activities to further develop the common good. CPI focuses on the individual’s intrinsic interest in pursuing commonly held public values such as equity, concern for future generations, and accountability. COM refers to the intensity to which people identify with the wants and the needs of others. Finally, SS involves the degree to which people are prepared to help and support others and the community as a whole, even at the expense of their personal benefits and reward ethics (Kim et al., 2013).

These four dimensions of PSM are grounded in either affective or non-affective motives (Perry and Wise, 1990; van Witteloostuijn et al., 2017). Perry and Wise (1990) make a distinction between rational, normative, and affective motives. The ability to influence public policy as a source of personal gain or considering public policy as a means to boost one’s self-esteem are rational motives that can serve as an incentive to engage in public services (Ritz, 2011). These rational, self-serving motives are reflected in the dimension of ***APM*** (Perry, 1996). Typical APM-motivated people seek status, power, and wealth for their own security and convenience (Ritz, 2011). APM is PSM’s core dimension for many behavioral outcomes of politicians, and is related to a power-based Machiavellian understanding of politics (Ritz, 2011, 2015). APM has many similarities with the need for power.

The need for power stems from a person’s desire to influence, teach or encourage others, and this mainly to demonstrate one’s own superior capabilities vis-à-vis others (Winter, 2010a). Power-motivated employees have good leadership abilities (House and Howell, 1992), excel in (organizational) politics (Winter, 2010b; Blickle et al., 2018), and flourish in hierarchically structured organizations in which they aim for high-level positions so that they can control the direction in which their company or organization is moving (McClelland and Boyatzis, 1982). Kumar and Beyerlein (1991) showed that need for power is positively associated with political activity, and Winter (2005, 2010a) demonstrated that power motivation scores of American presidents were significantly related to historians’ ratings of presidential greatness and the making of “great” decisions. Similarly, House et al. (1991) found that measures of presidential charisma were positively related to the president’s need for power. Tying these insights together, we expect that the individual’s willingness to participate in policy-making processes relates her or his need for power. Because typical APM behavior is most likely simultaneously intrinsically (e.g., having a good discussion on politics is in itself rewarding) as well as extrinsically rewarding (e.g., appraisal by other people) for power-motivated people, we expect that APM is linked to both implicit and explicit need for power.

H1a: APM is positively related to the explicit need for power.

H1b: APM is positively related to the implicit need for power.

Both ***COM*** and ***SS*** are affectively grounded, stemming from a motivation to help others and society (Perry, 1996; van Witteloostuijn et al., 2017). Both power and affiliation motives are often linked to pro-social and helping behaviors (Hofer et al., 2007). However, the underlying drivers differ substantially between both motives. That is, power-motivated persons do not help others or do not act-prosaically, neither because of an innate desire to help others nor because this feels right. They rather help others to show their own supremacy and competence. They help others to show that they are better in something, to show their supremacy, and to be admired afterward (Aydinli et al., 2014, 2016). However, for power-motivated people, neither self-sacrifice nor compassion is likely to be the underlying driver of their helping behavior. Quite to the contrary, compassion and self-sacrifice may even thwart the power motive, as they can be interpreted as signals of weakness and submission (Schultheiss et al., 2005).

Thus, we expect that high power may translate into low affectively grounded PSM motives COM and SS. Further, because typical COM and SS behaviors have most likely simultaneously task-intrinsic (e.g., the act of helping) as well as extrinsic incentives (e.g., being blamed from being weak) for power-motivated people, we expect similar relationships for explicit and implicit need for power.

H2a: COM is negatively related to the explicit need for power.

H2b: COM is negatively related to the implicit need for power.

H2c: SS is negatively related to the explicit need for power.

H2d: SS is negatively related to the implicit need for power.

Contrary to power, the need for affiliation is strongly connected to the desire to help others, and for creating and maintaining social bonds with others (Sokolowski and Heckhausen, 2008). Affiliation-motivated people help and listen to others because this creates a social bond between them. Hence, we expect that both COM and SS will connect positively with the need for affiliation. Yet, we expect differences in their relations with implicit and explicit affiliation. SS-motivated people are likely to engage in activities that are not *per se* beneficial to the self. SS-motivated behavior is not reward-seeking behavior (Perry, 1996). SS instigates “have-to” rather than “want-to” behavior. Indeed, SS-motivated behavior is stirred by sentiments of civic duty (Brewer et al., 2000; Roh et al., 2016). For example, Brewer and Selden (1998) found that whistle-blowers in the public sector are more motivated to perform their duties, placing more importance on self-sacrifice.

As discussed earlier, the presence of social-extrinsic incentives such as a call upon one’s civic duties is a necessary condition for explicit motives to become activated and to influence behaviors and perceptions (Stanton et al., 2010). Therefore, we expect a positive relation between explicit affiliation and SS. Even though the result of self-sacrificing may be beneficial or satisfying for the self, the act of sacrificing is in itself not pleasant. Thus, in terms of motives, the absence of task-intrinsic incentives makes that a necessary condition is not met for implicit motives to become aroused, and to affect behavior and perceptions (Stanton et al., 2010). Thus, we do not expect that implicit affiliation relates to SS.

H3a: SS is positively related to the explicit need for affiliation.

In contrast, COM reflects true altruistic motivations, characterized by high empathy (Perry, 1996). For example, Hsieh et al. (2012) found that COM-motivated individuals are more likely to engage in emotional efforts that help them place themselves in clients’ shoes and take clients’ interests to heart. This so-called “deep acting” is rewarding in itself and is fostered by a genuine inner urge to help others (Kanov et al., 2004; Hsieh et al., 2012), thus reflecting a want-to as well as a have-to behavior. For this reason, we expect that COM is both channeled by implicit and explicit motives.

H3b: COM is positively related to the explicit need for affiliation.

H3c: COM is positively related to the implicit need for affiliation.

Finally, ***CPI*** reflects an individual’s sense of obligation to the society in which s/he lives. For CPI-motivated people, serving the public feels as a duty one has to fulfill toward one’s government and community (Perry, 1996). CPI refers to actions generated to conform with social norms, and fits with values such as nationalism and loyalty to the country (Perry and Wise, 1990). Moreover, CPI reflects collectivist values and communitarian norms that favor the community over the individual. Hence, we expect that CPI is related to the need for affiliation.

The development of commitment in general and commitment to the public interest in specific requires a long socialization process. A first notion of what commitment is may already be acquired early in life through socialization through parents and close family (Grusec, 1992). Yet, educational programs, and peer and normative pressure make that the notion and importance of commitment is heavily reshaped and transformed later in life (Malerstein and Ahern, 1979; Alexander et al., 1990). As commitment mirrors the development process of explicit motives (McClelland and Pilon, 1983; Kasser et al., 2002), we expect a positive association with the explicit, but not with the implicit motives. Also, the strong sense of obligation to serve the group and the strong normative “have-to” character of CPI represent social-extrinsic incentives, strengthening our expectation that CPI is related to the explicit, rather than to the implicit need for affiliation.

H4: CPI is positively related to the explicit need for affiliation.

## Materials and Methods

We use two samples of undergraduate business students at a Belgian and a Dutch university. After excluding unmatched surveys and those with missing data, 165 valid observations were collected: 99 are third-year students of a Bachelor degree program in Business and Economics a in the Netherlands, and 66 are first-year Master students in Business and Economics in the Dutch-speaking part of Belgium. Undergraduate students are regularly used in Public Administration, being well suited to test the relation between core personality characteristics and PSM (see, e.g., Perry, 1996; Christensen and Wright, 2011; van Witteloostuijn et al., 2017; Ballart and Rico, 2018). Even though the parental socialization process in general and the profession of someone’s parents in particular impact a person’s level of PSM (Anderfuhren-Biget, 2012), students have limited work experience, which makes their PSM less affected by context due to work socialization and other sources of job-related variance (van Witteloostuijn et al., 2017).

After reading and signing an informed consent, we administered an explicit motives questionnaire and implicit motives test. Students then took a break of about 15 min after which they completed the PSM survey (including demographic items). When independent and dependent variables are administered by means of self-reported questionnaires, common-method variance (CMV) may bias regression analyses (Chang et al., 2010). In order to minimize possible effects of CMV, we separated both measures by means of short break (Podsakoff et al., 2012). Also, the implicit motives test was positioned in between the explicit motives and the PSM questionnaires, assessed by means of an indirect, computerized response latency task. This may also have reduced the likelihood of CMV.

We measured *explicit motives* with the achievement, affiliation, and power subscales of the Personality Research Form (PRF: Jackson, 1984), which is a self-report inventory of basic motives. Participants were asked to what extent each statement fitted with who they are. Sample items of the subscales are: “The ability to be a leader is very important to me” (power subscale); “I truly enjoy myself at social functions” (affiliation subscale); and “I often set goals that are very difficult to reach” (achievement subscale). Each subscale consists of sixteen seven-point Likert-type items with anchors 1 = “Fits not at all” and 7 = “Fits very well”. For each subscale, we calculated the individual measures as the mean score of the items, high scores indicating a good fit between the motive and the participant. Each subscale showed satisfactory to good internal consistency (Cronbach’s α) (PRF achievement: α = 0.79; PRF affiliation: α = 0.87; PRF power: α = 0.88).

*Implicit motives* have to be captured with indirect measures. Until recently, only projective methods such as the Thematic Apperception Test (TAT) and the Picture-Story Exercise (PSE) were available to assess implicit motives. A typical PSE or TAT consists of four to six pictures that depict people in a variety of social settings. For each picture, participants write an imaginative story. The assumption is that the content of the stories reflects people’s implicit motives (McClelland et al., 1989; Schultheiss and Brunstein, 2001). The content of these imaginative stories must be coded according to motivational coding schemes, empirically derived and refined over decades (see e.g., Smith, 1992; Winter, 1994). Test validity work confirms the good psychometric qualities of these measures (Pang, 2010). However, these measures are very cumbersome and extremely time-consuming for both participants and scholars.

The most interesting alternative to the PSE and TAT is the Implicit Association Test (IAT: Greenwald et al., 1998). The IAT is by far the most popular implicit measure (De Houwer and De Bruycker, 2007; Bar-Anan and Nosek, 2014). The IAT is a computerized response latency task designed to measure the relative strength of associations between two pairs of contrasted concepts (e.g., “pleasant–unpleasant” and “sunshine–rain”). Participants view stimuli that are representative of any of the four concepts one at a time on the computer screen, and categorize those stimuli by means of two response keys. An IAT consists of several rounds (“blocks”), each with several categorization tasks (“trials”) and specific instructions. For example, in the first block, participants may be instructed to “press the ‘e’ key for stimuli that are representative of ‘pleasant’ or ‘sunshine’ and press the ‘i’ key for stimuli that are representative of the ‘unpleasant’ or ‘rain’ category.” Afterward, in the second block, instructions may change to “press the ‘e’ key for stimuli that are representative of ‘unpleasant’ or ‘sunshine’ and press the ‘i’ key for stimuli that are representative of the ‘pleasant’ or ‘rain’ category.” If two concepts are highly associated (e.g., “sun” and “pleasant”), categorizations of associated stimuli take less time when the two associated concepts share the same response key than when they do not.

Still, administering an IAT is quite time-consuming, requiring a substantial time investment from each participant. This is why we developed a *brief* IAT, or BIAT. Unlike the IAT, which requires participants to focus simultaneously on the two pairs of contrasted concepts, the BIAT presents only two of the four concepts at a time (say, “sunshine” and “pleasant” as the focal concepts) and has simplified instructions. The instructions direct participants to press the same response key whenever an exemplar of one of the two focal concepts is presented and to press another key “for anything else.” These simplifications lead to substantial reduction in the time length of the task. Despite these modifications, the BIAT retains the favorable psychometric properties of the original IAT (Sriram and Greenwald, 2009; Bar-Anan and Nosek, 2014; Nosek et al., 2014). Recently, and important for our research, Hermans et al. (2017) and Slabbinck et al. (2018) optimized the BIAT for the assessment of implicit motives.

We assessed implicit motives by adopting the BIAT procedure of Slabbinck et al. (2018). We constructed one BIAT for each implicit motive. Each BIAT consists of five blocks. The first block (12 trials) is a practice block that acquainted participants with the task instructions, and the remaining four (20 trials each) are the critical blocks we used to calculate the participants’ implicit motives. In each trial, participants focused simultaneously on two labels that were shown one under the other on the top center of the computer screen. One label represented the focal concept [i.e., “Power” (Power BIAT), “Successful” (Achievement BIAT), or “Together” (Affiliation BIAT)], and the other label the focal attribute (i.e., “Pleasant”). Participants saw stimuli that were or were not representative of the focal concept and attribute one at a time on the center of the computer screen, pressing the “i” key as quickly as possible when the stimuli represented any of the focal categories and the “e” key when the stimuli were not representative of the focal categories. We derived BIAT scores – e.g., the BIAT score of “Power”–“Pleasant” relative to “No Power”–“Pleasant” – by comparing participants’ performance in two blocks that presented the same focal attribute (e.g., “Pleasant”), but with two different focal concepts (“Power” and “No Power”). For a more detailed explanation, we refer to Table 1.

**TABLE 1:** Structure of the BIATs **(Panel A)** and used stimuli **(Panel B)**.

(A) Structure of the BIATs							
				Stimuli belonging to the focal categories		Stimuli not belonging to the focal categories	
Block	# Trials	Focal concept label	Focal attribute label	Press the “e” key		Press the “i” key	
1. Practice	12	Positive Motive Label (1)	Pleasant	Positive Motive Stimuli (3)	Nice, Friendly, Pleasant, Lovely,	Negative Motive Stimuli (4)	Creepy, Nasty, Annoying, Undesired
2. Critical	20	Negative Motive Label (2)	Pleasant	Negative Motive Stimuli (4)		Positive Motive Stimuli (3)	
3. Critical	20	Positive Motive Label (1)	Pleasant	Positive Motive Stimuli (3)		Negative Motive Stimuli (4)	
4. Critical	20	Negative Motive Label (2)	Pleasant	Negative Motive Stimuli (4)		Positive Motive Stimuli (3)	
5. Critical	20	Positive Motive Label (1)	Pleasant	Positive Motive Stimuli (3)		Negative Motive Stimuli (4)	

**(B) Words that were used in each BIAT as focal labels and stimuli**							

	**Positive Motive Label**		**Negative Motive Label**	**Positive Motive Stimuli**		**Negative Motive Stimuli**	

Power BIAT	Power		No power	Control, Leader, Status, Authority		Submissive, Powerless, Impotence, Obedient	
Achievement BIAT	Successful		Unsuccessful	Perseverance, Ambitious, Curious, Competent		Give up, Being lazy, Resigned, Incompetent	
Affiliation BIAT	Together		Alone	Social, Connected, Together, Community		Solo, Independent, Individual, Hermit	

*We counterbalanced the order of focal concept labels so that half the participants started with “Power” as the focal concept label. We subtracted the mean response latency on the No Power–Pleasant blocks from the mean response latency on the Power–Pleasant block, and divided the result by the pooled standard deviation of the response latencies on all blocks to construct the BIAT scores (Greenwald et al., 2003). Positive scores indicate that participants associated “Power” with “Pleasant” more than with “Unpleasant.”*

Following Nosek et al. (2014), we used data from both practice and critical blocks to compute BIAT scores. We recoded extreme latencies below 400 ms and above 10,000 ms to these boundaries, and we discarded the first four trials of each block. We calculated individual BIAT scores using the so-called D measure (Greenwald et al., 2003). Positive scores indicated that participants associated “Pleasant” with “Power” (Power BIAT), “Successful” (Achievement BIAT), and “Together” (Affiliation BIAT) more than with “No Power” (Power BIAT), “Unsuccessful” (Achievement BIAT), and “Alone” (Affiliation BIAT).”

We measure *Public Service Motivation* by using a multidimensional scale composed of four dimensions: APM, CPI, COM, and SS (Perry, 1996). We adopted van Witteloostuijn et al. (2017) scale. This scale is similar to the revised 12-item measure of PSM as proposed by Kim (2011), but with some items slightly rephrased so that they are suited for data collection with undergraduate students: α = 0.78 for the Overall PSM scale, α = 0.68 for COM, α = 0.62 for SS, α = 0.66 for APM, and α = 0.61 for CPI. While the internal consistency values are not particularly high for the sub-dimensions, they are broadly in line with the reliability estimates of PSM reported in previous studies (Kim, 2009; Anderfuhren-Biget, 2012; Slabbinck, 2012; van Witteloostuijn et al., 2017; Awan et al., 2020). All items use a seven-point Likert-type scale, ranging from 1 = “strongly disagree” to 5 = “strongly agree.”

Following prior work on PSM antecedents, we add two further *control variables*: the age of the participant (in years) and gender (coded 1 for females, and 0 for males). To control for possible cross-cultural differences, we included the university of the participant (coded 1 for Belgium, and 0 for Netherlands). Table 2 presents the descriptive statistics and bivariate correlations. The correlations between the implicit and explicit motives are low. This is consistent with earlier work, suggesting that implicit and explicit motives are indeed distinct constructs, in line with theory (Schultheiss et al., 2009; Köllner and Schultheiss, 2014). Furthermore, correlations between all independent variables are all low to moderate, without any extreme values.

**TABLE 2:** Descriptive statistics and bivariate correlations.

	M	SD	1	2	3	4	5	6	7	8	9	10	11
PSM	3.99	0.71											
APM	4.41	1.06	0.62**										
SS	3.34	0.98	0.76**	0.21**									
COM	4.42	0.93	0.65**	0.15	0.40**								
CPI	3.81	1.03	0.81**	0.34**	0.58**	0.37**							
E Ach	4.61	0.69	0.12	0.15	–0.01	0.07	0.11						
E Aff	4.94	0.78	0.11	0.00	0.02	0.20**	0.12	0.16*					
E Pow	4.55	0.77	–0.07	0.20**	−0.19*	–0.14	–0.11	0.41**	0.41				
I Ach	0.83	0.34	0.08	0.16*	0.04	0.05	–0.01	0.05	0.05	0.04			
I Aff	0.43	0.41	0.00	–0.04	0.00	0.07	–0.03	–0.02	–0.02	0.04	0.11		
I Pow	0.39	0.37	−0.15*	–0.07	–0.10	−0.21**	–0.07	–0.04	–0.04	0.12	0.32**	0.15	
Age	22.12	1.59	0.06	0.06	0.03	0.03	0.05	0.11	0.11*	0.17*	0.02	0.00	0.04

*p ≤ 0.05 and **p ≤ 0.01; E = explicit and I = implicit.

## Results

Because our dependent variables are metric measures, we use ordinary least square regressions with Heteroscedastic Consistent Standard Errors (HC3; Hayes and Cai, 2007). To assess multicollinearity, we first checked the variance inflation factors (VIF); the coefficients were all below 1.5 (maximum VIF-value: 1.30), indicating that multicollinearity should not be a concern (Hayes et al., 2012). Table 3 shows the results of five regression models. For Model 1, we regressed the Overall PSM score on the implicit and explicit motives, as well as the control variables age, gender, and university. The same independent variables were used for the subsequent four models, taking each time a different PSM dimension as the dependent variable: APM (Model 2), SS (Model 3), COM (Model 4), and CPI (Model 5).

**TABLE 3:** Regression results for Overall PSM, APM, CPI, COM, and SS.

	Model 1: Overall PSM			Model 2: APM			Model 3: SS			Model 4: COM			Model 5: CPI		
	B	SE	*t*-statistic	B	SE	*t*-statistic	B	SE	*t*-statistic	B	SE	*t*-statistic	B	SE	*t*-statistic
Constant	2.345	0.836	2.807**	2.336	1.287	1.815	2.839	1.178	2.41*	2.432	1.114	2.183*	1.788	1.185	1.509
University	0.37	0.133	2.789**	0.564	0.175	3.225**	0.514	0.184	2.791**	–0.065	0.168	–0.388	0.465	0.188	2.474*
Age	0.024	0.029	0.822	–0.016	0.048	–0.324	0.018	0.045	0.397	0.058	0.04	1.45	0.034	0.041	0.83
Gender	0.108	0.113	0.96	0.24	0.165	1.458	0.151	0.16	0.944	–0.129	0.153	–0.845	0.169	0.173	0.975
E Ach	0.138	0.097	1.427	0.101	0.141	0.715	0.094	0.125	0.75	0.121	0.122	0.988	0.238	0.142	1.681
E Aff	0.171	0.077	2.212*	0.05	0.105	0.478	0.14	0.112	1.25	0.241	0.105	2.299*	0.254	0.098	2.609**
E Pow	–0.151	0.079	–1.905	0.229	0.132	1.743	–0.311	0.109	−2.846**	–0.235	0.106	−2.217*	–0.29	0.122	−2.384*
I Ach	0.23	0.156	1.474	0.512	0.228	2.247*	0.126	0.234	0.537	0.333	0.255	1.307	–0.055	0.221	–0.247
I Aff	0.125	0.157	0.796	0.105	0.212	0.497	0.194	0.207	0.939	0.118	0.204	0.579	0.077	0.19	0.403
I Pow	–0.273	0.164	–1.672	–0.334	0.215	–1.553	–0.159	0.233	–0.684	–0.564	0.225	−2.506*	–0.037	0.235	–0.157
F_9,155_	2.396*			3.387**			2.084*			3.002**			2.12*		
*R*^2^	0.139			0.151			0.113			0.143			0.104		

Heteroscedastic Consistent SEs are reported (HC3: Hayes and Cai, 2007); *p ≤ 0.05 and **p ≤ 0.01.

Concerning the control variables, only the participant’s university is significant in most models. Compared to Dutch participants, Overall PSM, APM, SS, and CPI are all significantly higher for Belgian participants. Model 1 reveals a significantly positive relation between explicit affiliation and Overall PSM. Neither any other explicit motive, nor any implicit motive reaches significance. Interestingly, Models 2-4 reveal different patterns for different PSM dimensions. The results of Model 2 are not in line with Hypotheses 1a and 1b: neither explicit, nor implicit power are significantly related to APM. Instead, implicit need for achievement is positively and significantly associated with APM.

In line with Hypothesis 2a and 2b, we find that that both implicit and explicit power are negatively and significantly related to COM (see Model 3). The results in Model 2 further indicate that explicit, but not implicit power is negatively related to SS. Hypothesis 2c is thus supported, while Hypothesis 2d is not.

Contrary to Hypothesis 3a, Model 2 shows that explicit affiliation is not associated with SS. However, and in line with Hypothesis 3b, explicit affiliation is significantly and positively related to COM. Hypothesis 3c is not supported, as the parameter estimates of Model 4 do not reveal a significant relation between implicit affiliation and COM. Finally, Model 4 provides full support for Hypothesis 4: CPI is significantly and positively linked with explicit affiliation, but not with implicit association. Next to this, CPI is negatively and significantly linked to explicit power.

## Discussion and Conclusion

Explicit affiliation is the only basic motive that is significantly related to Overall PSM. People who explicitly aim to take care of others, trying to build, maintain, and restore social bonds, also seem to be more motivated to serve the public interest. Thus, we find support for the idea that PSM is rooted in affiliative, affective motives, but not for the idea that PSM is also rooted in rational or selfish motives (Perry and Wise, 1990; Perry et al., 2010). However, different patterns emerge for PSM’s subdimensions. For APM, we find a significant and positive link with implicit achievement. Thus, not the direct reward from exerting power, but the indirect reward through policy achievements seems to motivate people to engage in policy-making. It is apparently not the policy-making process itself, but the decisions and achievements that are the results of this process that implicitly motivate people to engage in policy-making. However, the execution of decisions that that are the outcomes of a policy-making process is typically slow, and may go through several stages before action is taken (Lindblom, 1968). As implicit, contrary to explicit, motives rather predict long-term behavior (McClelland and Boyatzis, 1982; McClelland and Pilon, 1983), this may explain why we only identify a relationship with an implicit motive.

The affective PSM motives SS and COM are both negatively related to power. As expected, SS is only related to explicit power, whereas COM is associated with both implicit and explicit power. Thus, inner (COM) and/or external (COM and SS) feelings of power seem to refrain power-motivated people from engaging in affectively motivated PSM. Contrary to COM-motivated PSM, SS-motivated PSM is apparently not fueled by someone’s explicit need for affiliation. Additionally, our evidence reveals that CPI is positively associated with explicit affiliation, and negatively related with explicit power. Apparently, actions and commitments to serve the public interest are inconsistent with personal power motivations, confirming the selfish character of power (Dubois et al., 2015).

In all, our first contribution is that we confirm that PSM is stirred by individual “deep” characteristics (van Witteloostuijn et al., 2017). Along its rapid growth in popularity, PSM research has been increasingly criticized on its conceptualization (Bozeman and Su, 2015). Researchers additively pile up related, yet slightly different concepts and measures. This proliferation contributes to problems of conceptualization, giving rise to an increasing scrutiny against PSM as an independent construct (Bozeman and Su, 2015; Schott et al., 2019). Too few studies examine the fundamental roots of PSM and how those relate to development of an individual’s PSM (Bozeman and Su, 2015). Our study partially addresses this ontological issue and reveals that different basic human motives affect each PSM dimension differently. The reported relations between implicit and explicit motives, on the one hand, and PSM (dimensions), on the other hand, serve as another proof that PSM has trait-like aspects, and thus is not a purely attitudinal concept Our second contribution, and perhaps of much greater importance, is that we provide consistent and comprehensive evidence that different basic human motives are at play in the formation of PSM. We did so by introducing a recently developed implicit measure, the BIAT (Slabbinck et al., 2018), into the Public Administration literature. The BIAT is highly flexible and easy to adapt for the measurement of many other personality and attitudinal constructs (Sriram and Greenwald, 2009; Slabbinck et al., 2018), opening a gateway to a wide variety of future research opportunities in the tradition of behavioral Public Administration.

Our findings are also relevant from a practitioner’s viewpoint. This is particularly the case because implicit and explicit motives are triggered by different types of incentives: Implicit motives become aroused and affect behaviors by task-intrinsic incentives, whereas explicit motives are triggered by social-extrinsic incentives (McClelland et al., 1989; Stanton et al., 2010). We found that implicit achievement is positively related to APM, whereas explicit need for affiliation is positively related to CC and CPI, and explicit power is negatively related to all facets of PSM, but APM. Hence, different motives should be addressed to foster different types of PSM. For example, to attract citizens to partake in policymaking, one should not only search for people who are motived by implicit need for achievement, but, and perhaps even more importantly, policy-making should also be portrayed as a task or job that is rewarding in and of itself. This can be done by portraying policy-making as a job or task that provides ample opportunities to learn and to surpass one’s own standard of excellence, as these are features that are particularly attractive for implicit achievement-motivated people (Brunstein and Heckhausen, 2008). In similar vein, if the goal is instigating self-sacrifice or boosting commitment to the public interest, one should rather stress the social-extrinsic rewards of PSM-related outcomes. For example, the idea that engagement in PSM-related tasks will provide social approval or calls upon an individual’s moral duties may be particularly effective strategies to foster SS or CPI, as these features are especially attractive for people who score high on explicit affiliation (Sokolowski and Heckhausen, 2008).

A complete discussion of reward preferences and incentives is beyond the scope of this article, but these implications may shed a new and refreshing light on the discussion whether intrinsic rewards provided by the nature of job may be more important than extrinsic rewards (Wright, 2007; Wright and Grant, 2010). Our implications suggest that not only the type of incentive, but also the individual’s implicit and explicit motive profile needs to be considered in selecting effective incentives to foster different facets of PSM. This, obviously, needs to be addressed in future research.

This study is not without limitations, of course, which point to additional future research opportunities. First, van Witteloostuijn et al. (2017) relied on the HEXACO model of personality, and report that Overall PSM is rooted in Honesty-Humility, Emotionality, Extraversion, and Openness to Experience, but not in Agreeableness. Our evidence only suggests a relation between explicit affiliation and Overall PSM. Basic human motives are not identical to personality traits (Winter et al., 1998). Yet, affiliation may be linked to Honesty-Humility and Emotionality, but, if at all, affiliation is most likely to also connect to Agreeableness. From that perspective, one should not expect a relation between explicit affiliation and PSM. These different patterns call for further research into the basic human motives in combination and interaction with personality traits as antecedents of PSM.

Besides personality theory, PSM research may also benefit from theories from other disciplines, an example being sociology. We heavily relied on McClelland’s human motives theory (McClelland et al., 1989). Yet, human motives, and definitely implicit motives, seem to have much in common with Bourdieu’s concept of “habitus.” Similar to implicit motives, Bourdieu defined habitus as an individual’s second nature that guides the individual’s thoughts, perceptions and behaviors in a deeper, pre-reflexive and unconscious way (Costa and Murphy, 2015). Also in line with implicit motives, habitus is shaped by social interactions that are already internalized during early phases of a person’s socialization process (Costa and Murphy, 2015). As different elements of a habitus impact the formation of someone’s personality (Pickel, 2005; Kaiser and Schneickert, 2016), it is most likely that the elements of a person’s habitus, either or not in interaction with implicit or explicit aspects of someone’s personality or motives, may also determine the formation of PSM and its different subdimensions. Yet, in order to determine if and how habitus and (implicit) motives impact the formation of PSM (and many other constructs in public administration research), more multidisciplinary research is required.

The assessment of PSM is not perfect, yet, as is witnessed by the generally rather low dimensional reliabilities. Indeed, the measurement of PSM in general (Bozeman and Su, 2015) and the often relatively low internal consistency estimates of its subdimensions in particular (Kim, 2009; van Witteloostuijn et al., 2017; Awan et al., 2020) are concerns that are shared by many PSM researchers. Low internal consistency of the PSM subdimensions not only poses a risk to the interpretation of the results of our and other studies, but it also illustrates the need for PSM researchers to move beyond the use of explicit, self-reported survey questions to assess PSM and its subdimensions. Scholars heavily invest in developing better measures of PSM (Perry et al., 2010; Grimmelikhuijsen et al., 2017). Yet, to the best of our knowledge, these efforts are limited to developing or improving explicit, questionnaire-type PSM measures.

However, next to basic motives and personality traits, PSM may have an implicit or unconscious side, too, which cannot be captured by means of traditional, direct measurement instruments. Thus, the development and validation of an implicit PSM measure is potentially a worthwhile investment. The research of Resh et al. (2019) only strengthens this believe. That is, in their effort to better understand when and under which conditions prosocial motivations translate into prosocial work behaviors in public and non-profit organizations, they assessed prosocial motivations with both an implicit and explicit measure. Their results show that the relationship between implicit prosocial motivations and prosocial work behavior is more difficult to distort (e.g., by providing monetary incentives) than the relationship between explicit prosocial motivations and prosocial work behavior. In a similar vein, the development of an implicit PSM measure may thus open a whole new avenue of research in PSM research, exploring the relationship between explicit and implicit PSM (and its dimensions), and the potentially differential impact on a wide array of attitudes and actions (Slabbinck et al., 2018; Marvel and Resh, 2019; Resh et al., 2019).

We used a cross-sectional design to empirically test relationships that are theoretically causal. So, we cannot make any causal claims on the basis of our empirical evidence. Of course, a better way to examine the causal relationship between implicit motives and outcomes (here, PSM) is to use a longitudinal design (Antonakis et al., 2010). Such a design would not only yield better insights into the relations between core basic motives and personality traits, on the one hand, and PSM, on the other hand, but would also provide insights into the stability or malleability of (the relationship between) both implicit/explicit motives and (the dimensions of) PSM. Note that, given the extant evidence regarding the relative stability of core human motives and personality traits, the claim that causality runs from these constructs to PSM, rather than the other way around, is theoretically plausible.

Another issue involves the measurement of PSM and its relation to behavior. How is the survey measure of PSM related to actual behavior? Here, the evidence is mixed. For instance, Esteve et al. (2016) report results indicating that the survey measure of PSM is positively associated with this individual’s public good investment in a laboratory experimental setting, but Awan et al. (2020) find that the survey measure of PSM is positively associated with reported, but not with actual blood donation. This suggests that further work is needed to examine the complicated web of (the absence of) relationships between survey measures of PSM (and the underlying dimensions), personality traits, explicit and implicit motives, and perceptual attitudes and actual behaviors.

A final remark relates to our sample. Our sample only includes bachelor and master students from only two countries. Future studies could seek to investigate whether or not the associations that we have found uphold in samples from other countries, and of different types of practitioners. For example, a future research opportunity is to analyze whether or not the effects of implicit and explicit motives on PSM and its dimensions vary with characteristics of national cultures, and/or the extent and nature of job experience. This aligns well with the plea for more replication in Public Administration, of both the exact and extended types (Walker et al., 2019).

## Data Availability Statement

The raw data supporting the conclusions of this article will be made available by the authors, without undue reservation.

## Ethics Statement

Ethical review and approval was not required for the study on human participants in accordance with the local legislation and institutional requirements. The patients/participants provided their written informed consent to participate in this study.

## Author Contributions

HS and AV equally contributed to the conception and design of the study, the data collection, and the statistical analysis. HS wrote the first draft of the manuscript. AV revised the manuscript. All authors contributed to manuscript revision, read and approved the submitted version.

## Conflict of Interest

The authors declare that the research was conducted in the absence of any commercial or financial relationships that could be construed as a potential conflict of interest.
